# A Phase I Randomized Trial of Once‐Daily Versus Twice‐Daily Recombinant Human Parathyroid Hormone (1‐84) for Hypoparathyroidism

**DOI:** 10.1002/jbm4.10758

**Published:** 2023-05-31

**Authors:** Steven W. Ing, Richard D. Finkelman, Ping He, Aliya A. Khan, Michael Mannstadt, Lars Rejnmark, Ivy Song, István Takács, Yuna Wu

**Affiliations:** ^1^ Division of Endocrinology, Diabetes, and Metabolism Ohio State University Wexner Medical Center Columbus OH USA; ^2^ Takeda Pharmaceuticals USA, Inc. Lexington MA USA; ^3^ Divisions of Endocrinology and Metabolism and Geriatrics McMaster University Oakville ON Canada; ^4^ Endocrine Unit Massachusetts General Hospital and Harvard Medical School Boston MA USA; ^5^ Department of Clinical Medicine – Department of Endocrinology and Internal Medicine Aarhus University and Aarhus University Hospital Aarhus Denmark; ^6^ Department of Internal Medicine and Oncology Semmelweis University Budapest Hungary

**Keywords:** DISORDERS OF CALCIUM/PHOSPHATE METABOLISM, HORMONE REPLACEMENT/RECEPTOR MODULATORS, PARATHYROID‐RELATED DISORDERS, THERAPEUTICS

## Abstract

Recombinant human parathyroid hormone (1‐84), rhPTH(1‐84), is an approved adjunctive treatment to oral calcium and active vitamin D for adult patients with hypoparathyroidism; however, there is limited information on the effect of twice daily (BID) dosing of rhPTH(1‐84). This was a phase I, open‐label, randomized, crossover, multicenter study conducted in adult patients with chronic hypoparathyroidism. The primary objective was to assess the pharmacokinetic profile and pharmacodynamic effects of 1 day of treatment with rhPTH(1‐84) administered subcutaneously at 25 μg BID, 50 μg BID, and 100 μg once daily (QD) with or without supplemental oral calcium. Safety and tolerability were evaluated as secondary objectives. In total, 33 patients with chronic hypoparathyroidism completed the study. Treatment with rhPTH(1‐84), both BID and QD, over the short‐term maintained serum calcium, lowered serum phosphorus, decreased urinary calcium excretion, and increased urinary phosphorus excretion. The decrease in urinary calcium excretion was numerically greater for BID than QD. Generally, baseline‐adjusted pharmacokinetic parameters including area under the curve and maximum observed concentration increased with increasing rhPTH(1‐84) dose, although this effect was not dose proportional. No new safety findings were observed. Our study revealed no differences thought to be clinically meaningful in pharmacokinetic or pharmacodynamic parameters with BID versus QD rhPTH(1‐84) dosing. Future long‐term studies are warranted to further elucidate the effects of alternative dosing strategies. © 2023 Takeda Development Center Americas, Inc and The Authors. *JBMR Plus* published by Wiley Periodicals LLC on behalf of American Society for Bone and Mineral Research.

## Introduction

Lack of parathyroid hormone (PTH) in hypoparathyroidism leads to reduced bone turnover, reduced calcium mobilization from bone, diminished renal production of active vitamin D, and loss of calcium‐conserving and phosphaturic effects on renal tubules, resulting in hypocalcemia, hyperphosphatemia, and hypercalciuria.^(^ ^1^, ^2^, ^3^ ^)^ Hypocalcemia associated with hypoparathyroidism is often managed via conventional therapy with oral calcium and active vitamin D; however, this therapy does not replace other physiologic functions of PTH.^(^ ^4^ ^)^ Compared with the general population, patients with chronic hypoparathyroidism whose condition is managed with calcium and active vitamin D alone are at increased risk of renal complications (eg, nephrolithiasis, nephrocalcinosis, and chronic kidney disease), cardiovascular conditions, urinary tract infection, immune system impairment, and hospitalization due to infection.^(^ ^5^, ^6^, ^7^, ^8^, ^9^, ^10^ ^)^ In addition, patients report high rates of neurologic and neuromuscular symptoms, including fatigue, cramping, paresthesia, mental lethargy, inability to concentrate, and forgetfulness.^(^ ^11^, ^12^, ^13^, ^14^ ^)^

Recombinant human parathyroid hormone (1‐84), rhPTH(1‐84), is an approved adjunctive treatment to oral calcium and active vitamin D for adult patients with hypoparathyroidism.^(^ ^15^ ^)^

The pivotal phase III, placebo‐controlled REPLACE study established the efficacy and safety of once daily (QD) administration of rhPTH(1‐84) at 50, 75, or 100 μg over 24 weeks in adults with chronic hypoparathyroidism.^(^ ^16^ ^)^ In REPLACE, 53% of patients in the rhPTH(1‐84) arm, compared with 2% of patients in the placebo arm, achieved the primary study endpoint (≥50% reduction from baseline in oral calcium and active vitamin D doses with maintenance of normal albumin‐corrected serum calcium, ideally within a target range of 8.0 to 9.0 mg/dL [2.0 to 2.25 mmol/L]; *p* < 0.0001). Urinary calcium excretion did not differ between the rhPTH(1‐84) and placebo arms, decreasing by −73.6 mg/24 hours and −83.8 mg/24 hours, respectively (*p* = 0.57). The efficacy of rhPTH(1‐84) QD was maintained during a 5‐year open‐label extension of REPLACE; in addition, mean urinary calcium excretion declined to within the reference range for both men and women by the end of the analysis period.^(^ ^17^ ^)^

A phase I, dose‐escalating study evaluating the pharmacokinetics (PK) and pharmacodynamics (PD) of QD rhPTH(1‐84) at 50 or 100 μg administered subcutaneously (SC), in patients with chronic hypoparathyroidism, found that the half‐life of PTH was approximately 2.5 to 3 hours with either dose; plasma PTH levels peaked at 10 to 15 minutes and then again at 1 to 3 hours after injection, and then declined to baseline by 12 to 24 hours posttreatment.^(^ ^18^ ^)^ Given the relatively short plasma half‐life of PTH after a single SC dose, investigators evaluated more frequent administration schedules.

Using a quantitative systems pharmacology modeling approach to simulate outcomes with varying rhPTH(1‐84) doses, Khurana and colleagues^(^ ^19^ ^)^ showed that 50 μg twice daily (BID) was associated with greater reductions in 24‐hour urinary calcium excretion than 100 μg QD dosing; both regimens maintained serum calcium within the reference range.

These previous studies motivated the investigation of BID versus QD dosing to compare these two dosing regimens for SC rhPTH(1‐84) administration. As a condition of the approval of rhPTH(1‐84) for hypoparathyroidism, the US Food and Drug Administration mandated the execution of a postmarketing trial to assess the PK and PD effects of rhPTH(1‐84) dose and dosing regimen on the control of serum and urinary calcium. The present study reports the effects of one day of treatment with rhPTH(1‐84) administered as 25 μg BID, 50 μg BID, and 100 μg QD, with and without supplemental calcium, on PK and PD outcomes in adults with chronic hypoparathyroidism.

## Patients and Methods

### Study design

**Fig. 1:**
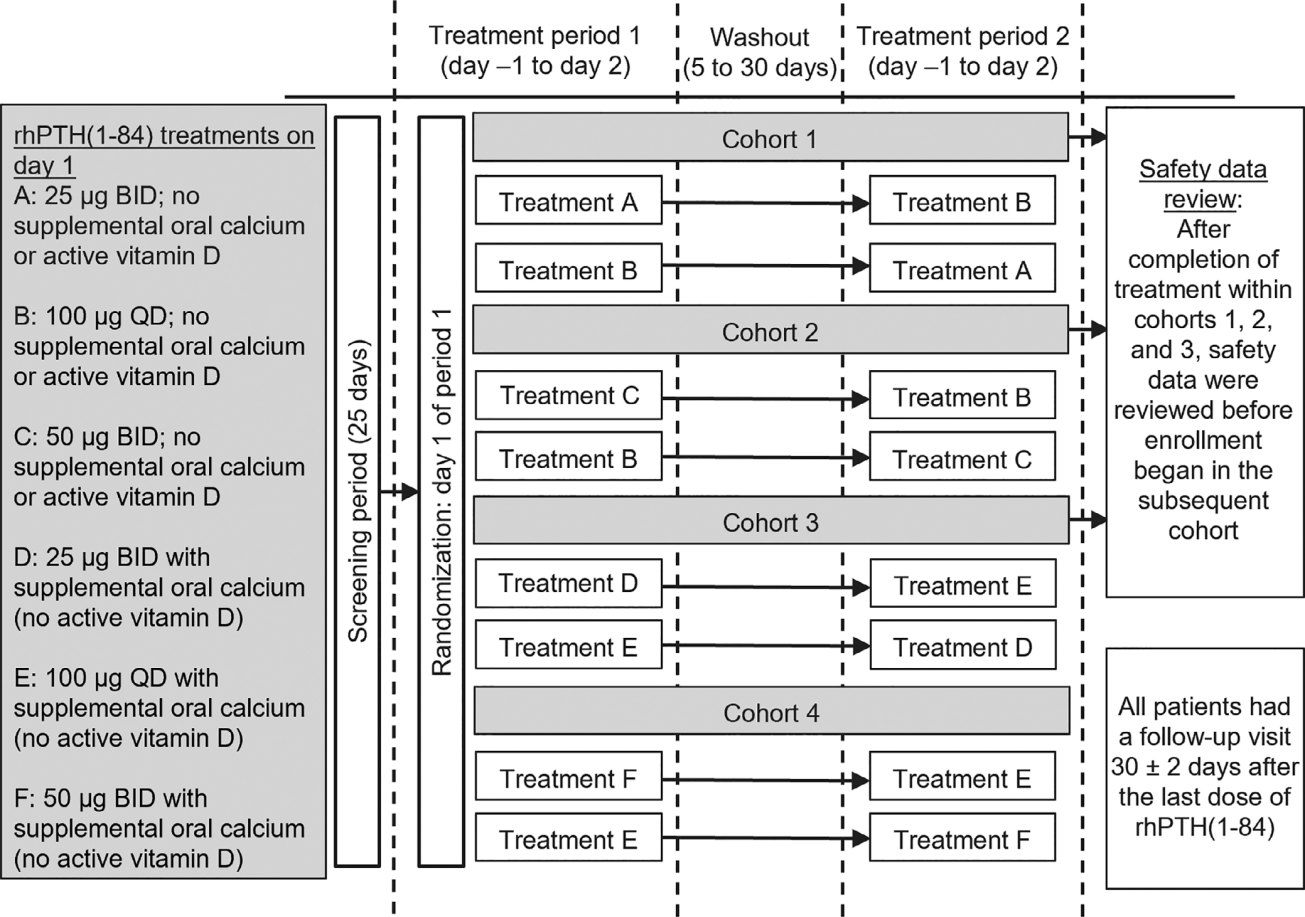
Study design. Patients were randomized sequentially into four treatment cohorts as illustrated and treated with QD or BID rhPTH(1‐84). Patients received a single‐day treatment in each of the two treatment periods, separated by a washout period of 5 to 30 days. Within each cohort, patients were randomly assigned 1:1 to treatment sequence order QD followed by BID or BID followed by QD. BID = twice daily; day −1 = the day before rhPTH(1‐84) dosing; day 1 = the day of rhPTH(1‐84) dosing; day 2 = the day following rhPTH(1‐84) dosing (ie, through 24 hours following the single QD rhPTH[1‐84] dose or the second BID rhPTH[1‐84] dose); QD = once daily; rhPTH(1‐84) = recombinant human parathyroid hormone (1‐84).

This was a phase I, open‐label, randomized, multicenter, sequential, four‐cohort, two‐period crossover study conducted in adults with chronic hypoparathyroidism (Clinicaltrials.gov identifier, NCT02781844; PARALLAX). The study was conducted at multiple centers across North America and Europe from May 2016 to May 2019. The study compared QD and BID dosing regimens of rhPTH(1‐84) without (cohorts 1 and 2) and with (cohorts 3 and 4) supplemental oral calcium and in the absence of administration of active vitamin D (eg, calcitriol; all cohorts) over a single day of treatment (Fig. 1). The primary objective was to assess the PK profile and PD effects (serum calcium, urinary calcium excretion) of rhPTH(1‐84) at doses of 25 μg BID, 50 μg BID, and 100 μg QD, as well as the effect of supplemental oral calcium intake. Secondary objectives were to assess the safety and tolerability of treatment with rhPTH(1‐84).

The study enrolled patients aged ≥18 years with a diagnosis of chronic hypoparathyroidism of ≥12 months' duration who required conventional therapy of ≥1000 mg/day oral calcium and ≥0.25 μg/day active vitamin D (calcitriol or equivalent). Included patients had serum calcium within the normal reference range (or not considered clinically significant by the investigator if outside of this range), urinary calcium excretion ≥200 mg (5 mmol)/24 hours, normal serum magnesium levels, and normal serum thyroid function tests before the initiation of study procedures. Patients with hypoparathyroidism resulting from an activation mutation in the *CASR* gene or impaired responsiveness to PTH (pseudohypoparathyroidism) were excluded from the study. Additional key inclusion and exclusion criteria are provided in Table 1.

**Table 1:** Additional Key Inclusion and Exclusion Criteria

Inclusion criteria	Serum 25(OH)D level between the lower limit of the reference range and 1.5 times the upper limit of the reference range at the clinical screening visit^a^
	Serum creatinine <1.5 mg/dL (<133 μmol/L) and estimated creatinine clearance >60 mL/minute at the clinical screening visit, and serum creatinine <1.5 mg/dL (<133 μmol/L) on day −2 of treatment period 1
Exclusion criteria	Any disease that could affect calcium metabolism or calcium‐phosphate homeostasis other than hypoparathyroidism, as determined by the investigator, including but not limited to: active hyperthyroidism; poorly controlled insulin‐dependent diabetes mellitus or type 2 diabetes mellitus; severe and chronic cardiac, liver, or renal disease; Cushing syndrome; neuromuscular disease such as rheumatoid arthritis; myeloma; pancreatitis; malnutrition; rickets; recent prolonged immobility; active malignancy, bone metastases, or a history of skeletal malignancies; primary or secondary hyperparathyroidism; a history of parathyroid carcinoma; hypopituitarism, acromegaly; or multiple endocrine neoplasia types 1 and 2
	Patients at increased baseline risk for osteosarcoma such as those with Paget disease of bone or unexplained elevations of alkaline phosphatase, patients with open epiphyses, patients with hereditary disorders predisposing to osteosarcoma, or patients with a prior history of external beam or implant radiation therapy involving the skeleton
	Use of thiazide diuretics within 14 days before rhPTH(1‐84) treatment in this study
	Treatment with rhPTH(1‐84), N‐terminal PTH, PTH‐related peptides or analogs, calcitonin, or cinacalcet hydrochloride, in the 3 months before rhPTH(1‐84) treatment in this study
	Dependence on regular parenteral calcium infusions (eg, calcium gluconate) to maintain calcium homeostasis within 3 months of enrollment Use of the following within 30 days of study entry: loop diuretics, lithium, systemic corticosteroids (per judgment of the investigator: stable doses of hydrocortisone [eg, as treatment for Addison disease] could be acceptable) Use of fluoride tablets, oral bisphosphonates, methotrexate, growth hormone, digoxin, raloxifene, or similar selective estrogen receptor modulators within 6 months prior to study entry Use of intravenous bisphosphonates or drug or alcohol abuse, as determined by the investigator, within 12 months of study entry

*Note*: rhPTH(1‐84) = recombinant human parathyroid hormone (1‐84).

^a^ Or not considered clinically significant by the investigator if outside of this range.

Enrollment in the study was staggered such that each treatment cohort was filled sequentially. After completion of treatment within each of cohorts 1, 2, and 3, safety data were reviewed before enrollment began in the subsequent cohort. Within each cohort, patients were randomly assigned 1:1 to treatment sequence order: QD followed by BID, or BID followed by QD. Randomization was achieved via allocation of a computer‐generated randomization number and assignment to a cohort via interactive response technology. Patients received a single day of treatment in each of the two treatment periods, separated by a washout period of 5 to 30 days. The QD dose or first BID dose of rhPTH(1‐84) was administered SC in the thigh in the morning after an overnight fast of ≥8 hours. For patients who received rhPTH(1‐84) BID, the second dose was administered in the opposite thigh 12 hours after the first dose. The day before rhPTH(1‐84) treatment was defined as day −1, the day of rhPTH(1‐84) treatment was defined as day 1, and the day after rhPTH(1‐84) treatment was defined as day 2. Patients continued to receive their routine supplemental calcium and active vitamin D through day −1 in each of the two treatment periods. On day 1, supplemental calcium was withheld for cohorts 1 and 2 only, and active vitamin D was withheld for all cohorts. Supplemental calcium and active vitamin D were resumed after the completion of all study procedures on day 2 (ie, 24 hours after the QD dose or second BID dose). All patients had a follow‐up visit which took place 30 ± 2 days after the last dose of rhPTH(1‐84) was administered.

### Assessments

Plasma PTH concentration was measured in blood samples collected predose; at 10, 20, 30, 60, and 90 minutes postdose; and at 2, 4, 8, 12, 16, and 24 hours postdose. Samples were analyzed for intact PTH by Celerion (Lincoln, NE, USA), using a previously validated second‐generation immunoradiometric assay with a quantification range of 10 to 2000 pg/mL (Whole PTH™ [1‐84] Specific kit; Scantibodies Laboratory, Inc., Santee, CA, USA).

PD endpoints included albumin‐corrected serum calcium, serum phosphorus, and serum creatinine measurements; serum calcium‐phosphate product was calculated based on albumin‐corrected serum calcium and serum phosphate concentrations. Total urinary calcium, phosphorus, sodium, magnesium, citrate, and cyclic adenosine monophosphate (cAMP) excreted over 24 hours postdose (both absolute concentrations and concentrations relative to total urinary creatinine excreted over 24 hours postdose) were also calculated and were assayed using the most current validated bioanalytical methods according to the relevant standard operating procedures in place at PPD Central Laboratory (Highland Heights, KY, USA), and at inVentiv Health Clinique Inc. (Quebec, QC, Canada). Total 24‐hour urine assessments were calculated by summing the individual measurements from each component collection period (collection periods relative to the first dose: −24 to −21 hours, −21 to −18 hours, −18 to −15 hours, −15 to −12 hours, −12 to −9 hours, −9 to −6 hours, −6 to −0 hours, 0 to 3 hours, 3 to 6 hours, 6 to 9 hours, 9 to 12 hours, 12 to 15 hours, 15 to 18 hours, and 18 to 24 hours). Fractional excretion of calcium, phosphorus, and magnesium were evaluated in each collection period and calculated as follows: *100 × (urine concentration of the analyte × serum creatinine]/[serum concentration of the analyte × urine creatinine)*. The serum concentration values used for the calculation of fractional excretion values were those at, or closest to, the middle of the urine collection period. Blood samples for PD endpoints were scheduled at 24, 22.5, 20, 18, 16, 14, 12, 10.5, and 8 hours before the first dose; predose on day 1; 1.5, 4, 6, 8, 10, 12, 13.5, 16, 20, and 24 hours postdose on day 1; and continuing through day 2 for BID dosing. Urine for 24‐hour urinary calcium excretion analysis was collected continuously on day −1 and on treatment day 1 through day 2.

Treatment‐emergent adverse events (TEAEs) were recorded continuously from the time of administration of the first rhPTH(1‐84) dose through study completion. TEAEs were defined as any unfavorable and unintended sign (including an abnormal laboratory finding), symptom, or disease temporally associated with use of the study drug, regardless of whether the event was considered by the clinical study investigator to be related to the study drug. Serious TEAEs were defined as any adverse event that required hospitalization or prolonged an ongoing hospitalization, caused persistent or significant disability, was considered by the investigator to be an important medical event, was life‐threatening, or resulted in death.

### Data analyses

The safety analysis set included all patients who received one or more doses of rhPTH(1‐84). The PK analysis set comprised patients who received one or more doses of rhPTH(1‐84) and had one or more evaluable postdose PK concentrations available for one or more dose regimens. The PD analysis set comprised patients who received one or more doses of rhPTH(1‐84) and had one or more evaluable postdose PD measurements available for one or more dose regimens. PK parameters included area under the curve (AUC), the maximum observed concentration reported (C_max_), and the t_½_ (half life). PK parameters were determined from plasma concentration‐time data by noncompartmental analysis using WinNonlin Phoenix version 8.1 (Pharsight Corporation, Mountain View, CA, USA). PD parameters, including AUC from time 0 to 24 hours after the first rhPTH(1‐84) dose (AUC_0–24_), were calculated from raw serum concentrations using WinNonlin Phoenix version 8.1.

In total, eight patients were required to complete each treatment in each cohort. The sample size was determined based on a similar previous PK/PD study.^(^ ^18^ ^)^ The number of participants was not based on statistical power considerations because the statistical analyses were primarily descriptive and hypothesis testing was not prespecified in the study. Continuous variables were summarized with descriptive statistics as mean ± SD and categorical variables as number and percentage. A post hoc statistical analysis was performed to compare the effects of BID versus QD rhPTH(1‐84) administration on urinary calcium excretion. Urinary calcium and urinary calcium relative to creatinine change from baseline as well as percentage change from baseline were calculated for 50 μg BID and 100 μg QD for day 1 to 2 and compared between 50 μg BID and 100 μg QD using a two‐sample *t* test, for patients in cohorts 2 (not receiving supplemental calcium) and 4 (receiving supplemental calcium).

### Ethics

The study protocol and all‐subject consent form were reviewed and approved by the institutional review board/independent ethics committee and regulatory agency, as appropriate, before study initiation. The study was conducted in accordance with the International Conference on Harmonization of Good Clinical Practice guideline, the Declaration of Helsinki, and other applicable local requirements. All patients provided written informed consent before any study procedure was performed.

## Results

### Patient characteristics

**Fig. 2:**
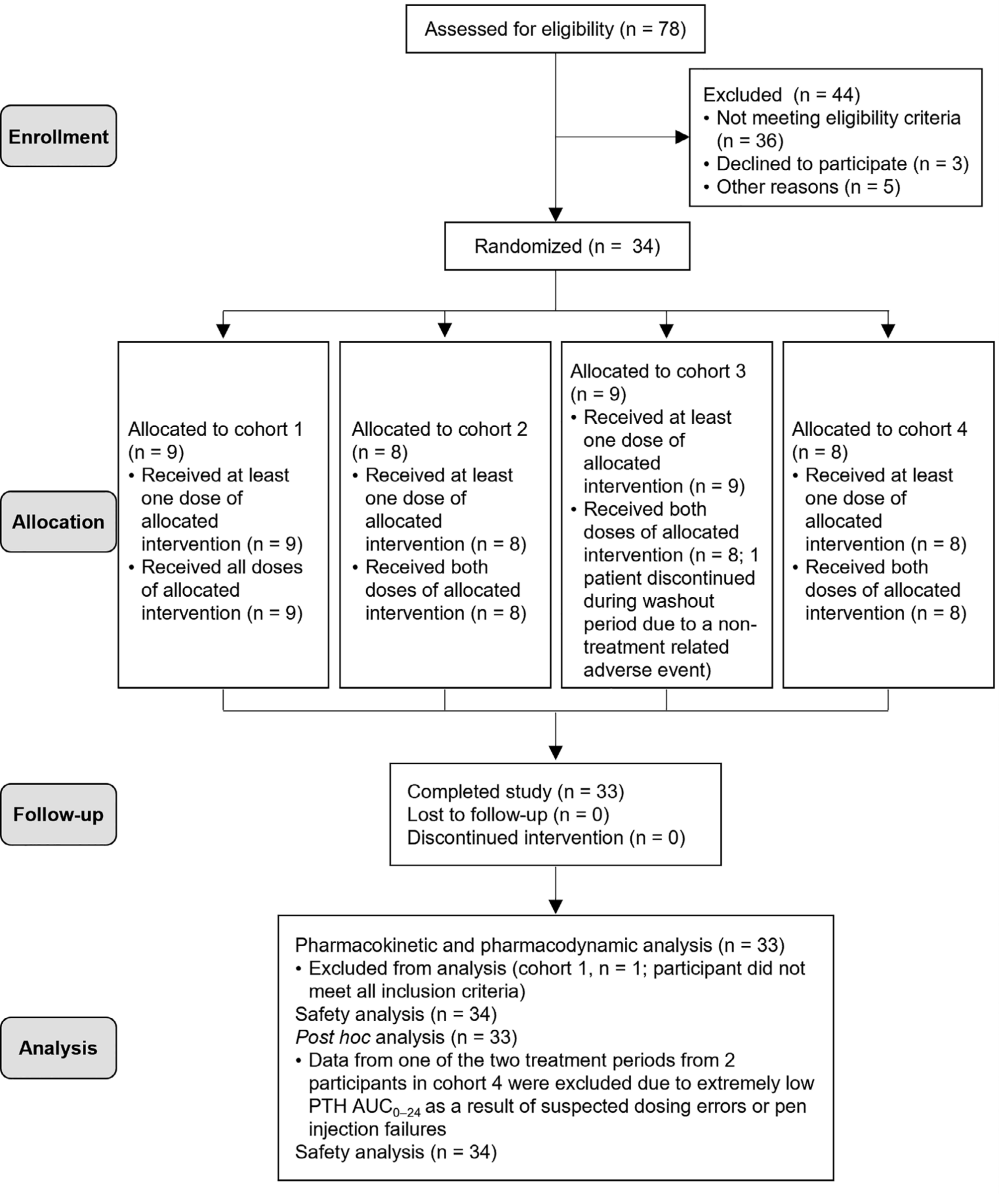
Patient disposition flow chart. PTH AUC_0–24_ parathyroid hormone AUC_0‐24_ = area under the curve from time 0 to 24 hours after the first rhPTH(1‐84) dose; rhPTH(1‐84) = recombinant human parathyroid hormone (1‐84).

Out of 78 screened patients, a total of 34 patients were enrolled (Fig. 2), of whom 33 (97.1%) had a diagnosis of chronic hypoparathyroidism and completed the study. One patient in cohort 3 who received rhPTH(1‐84) 100 μg QD withdrew from the study during the washout period after treatment period 1 because of a TEAE (not related to study treatment per the investigator) reported as exacerbation of depression. All 34 enrolled patients were included in the safety analysis set. The PK and PD analysis sets each comprised 33 patients. The data from one patient in cohort 1 were excluded from PK and PD analyses for failure to meet an inclusion criterion because this patient was considered not to have hypoparathyroidism. Demographics and clinical characteristics of patients are summarized in Table 2. At baseline, the mean body mass index (BMI) of patients in cohort 2 was lower than in other cohorts. Of the 4 cohorts, patients in cohort 2 also had the lowest baseline oral calcium and active vitamin D doses.

**Table 2:** Demographics and Baseline Clinical Characteristics

Parameter	Cohort 1 (*n* = 9)	Cohort 2 (*n* = 8)	Cohort 3 (*n* = 9)	Cohort 4 (*n* = 8)	All (*N* = 34)
Age (years), mean (SD)	42.6 (9.1)	48.9 (13.5)	52.4 (13.3)	45.8 (9.0)	47.4 (11.5)
Female, *n* (%)	9 (100)	6 (75.0)	8 (88.9)	7 (87.5)	30 (88.2)
Race, *n* (%)					
White	8 (88.9)	8 (100)	8 (88.9)	8 (100)	32 (94.1)
Black/African American	0	0	1 (11.1)	0	1 (2.9)
Multiple	1 (11.1)	0	0	0	1 (2.9)
BMI (kg/m^2^), mean (SD)	34.1 (11.5)	27. 6 (5.2)	33.1 (7.0)	32.9 (13.0)	32.03 (9.6)
Calcium dose (mg/day), mean (SD)	1884 (1409)	1441 (324)	3072 (3278)	2583 (1093)	2259 (1941)
Active vitamin D dose (μg/day), mean (SD)^a^	1.00 (1.16)	0.59 (0.27)	0.92 (0.49)	0.69 (0.32)	0.81 (0.67)

^a^ Calculated as calcitriol equivalents.

### PK

**Fig. 3:**
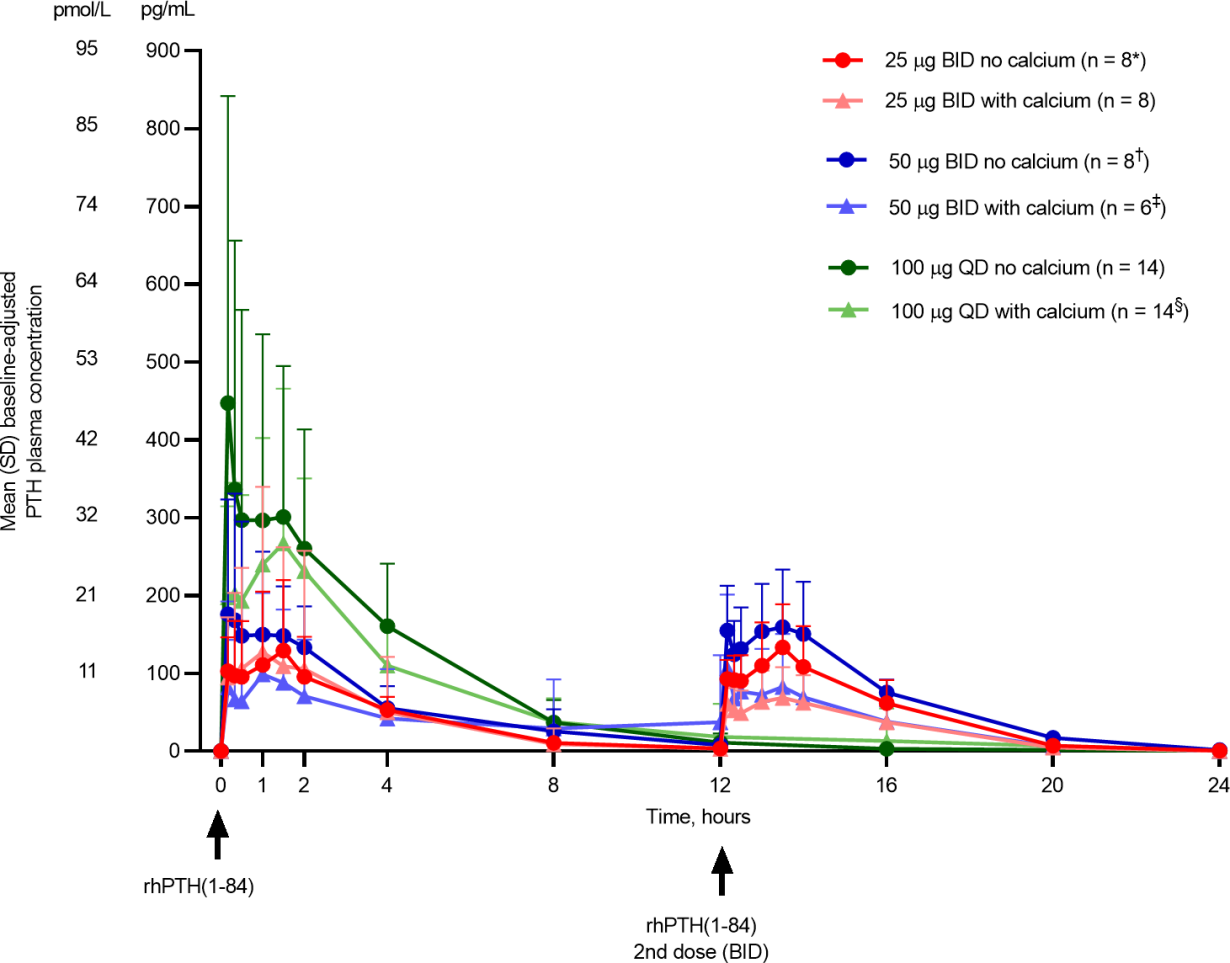
Baseline‐adjusted PTH plasma concentration. Data for 100 μg QD without calcium supplementation are from cohorts 1 and 2, and data for 100 μg QD with calcium supplementation are from cohorts 3 and 4. Error bars represent SD. *n = 7 at 16 hours. ^†^ *n* = 7 at 4 and 12.5 hours. ^‡^ *n* = 5 at 20 and 24 hours. ^§^ *n* = 11 at 2 hours; *n* = 12 at 16 hours; *n* = 13 at 1.5, 8, and 12 hours. BID = twice daily; QD = once daily; rhPTH(1‐84) = recombinant human parathyroid hormone (1‐84).

A double PTH peak concentration‐time profile was observed for all dose regimens, with an initial peak occurring at 5 to 30 minutes after injection and a second peak approximately 1 to 2 hours later. Following achievement of peak levels, mean concentrations of PTH declined steadily and became undetectable at 12 to 24 hours postdose (Fig. 3). Following the second administration of rhPTH(1‐84) for BID dosing, mean plasma PTH concentrations increased, peaking at approximately 13 to 14 hours after the initial dose and gradually decreasing thereafter. In general, baseline‐adjusted PTH PK parameters including AUC_0–24_ and C_max_ increased as the rhPTH(1‐84) dose increased (Table 3). However, dose proportionality was not observed, likely because of high PK variability within patients, within cohorts, and across cohorts. The t_½_ of PTH was comparable across dose regimens and ranged from 1.5 hours for rhPTH(1‐84) 25 μg BID with calcium to 2.2 hours for 100 μg QD with calcium.

**Table 3:** Baseline‐Adjusted PTH PK Parameters According to Treatment Group

	rhPTH(1‐84) treatment											
	No calcium						With calcium					
	25 μg BID (*n* = 9)		50 μg BID (*n* = 8)		100 μg QD (*n* = 17)		25 μg BID (*n* = 8)		50 μg BID (*n* = 8)		100 μg QD (*n* = 17)	
Parameter	Value	*n*	Value	*n*	Value	*n*	Value	*n*	Value	*n*	Value	*n*
C_max_ (pg/mL)	137 (50.9)	8	214 (51.6)	8	339 (138)	15	121 (83.6)	8	101 (106)	6	241 (69.6)	14
AUC_0‐24_ (hours · pg/mL)	945 (29.9)	8	1358 (31.3)	8	974 (335)	15	578 (94.3)	8	606 (135)	6	1107 (59.3)	13
t_½_ (hours)	1.74 (39.5)	4	2.07 (38.7)	7	1.97 (42.4)	13	1.45 (62.3)	3	1.77 (−)	1	2.19 (38.2)	12

*Note*: Values are given as geometric mean (CV% of geometric mean) unless otherwise stated. A patient in cohort 1 (25 μg BID, no calcium; 100 μg QD, no calcium) was not included in PK analyses for not meeting inclusion criteria. Data for 1 participant receiving 100 μg QD with supplemental oral calcium were excluded from all PK summary tables and listings as PK parameters except for C_max_ could not be calculated owing to the number of time points with missing data.

Abbreviation: AUC_0‐24_ = area under the curve from time 0 to 24 hours after the first rhPTH(1‐84) dose; BID = twice daily; C_max_ = maximum observed concentration; CV = coefficient of variation; *n* = number of observations for the analysis; PK = pharmacokinetic; QD = once daily; rhPTH(1‐84) = recombinant human parathyroid hormone (1‐84).

### PD

**Fig. 4:**
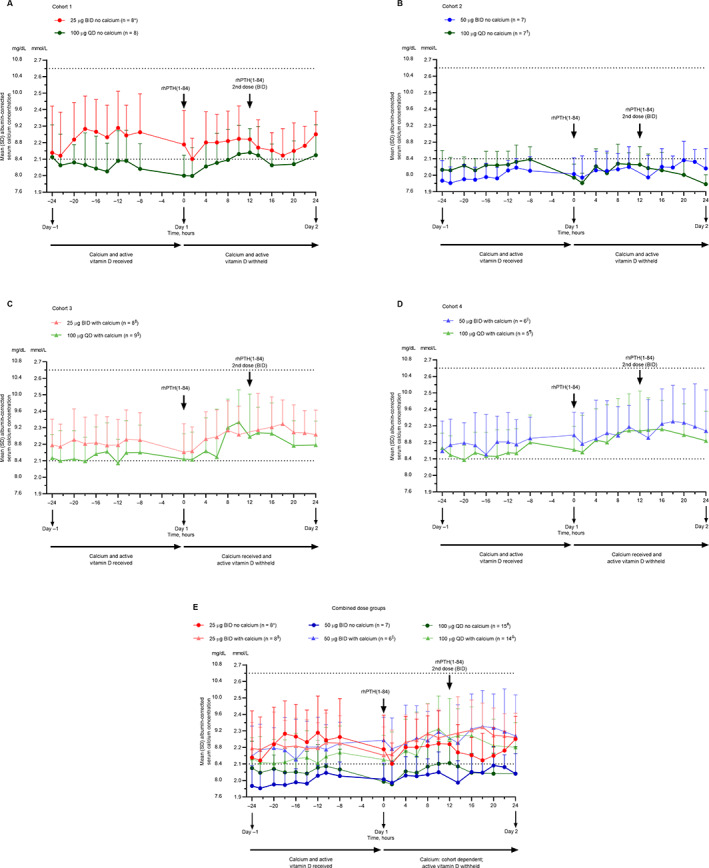
Mean albumin‐corrected serum calcium concentration in the study cohorts. (*A*) Cohort 1. (*B*) Cohort 2. (*C*) Cohort 3. (*D*) Cohort 4. (*E*) Combined dose groups (data for 100 μg QD without calcium supplementation are from cohorts 1 and 2, and data for 100 μg QD with calcium supplementation are from cohorts 3 and 4). Error bars represent SD. Horizontal lines indicate the reference range. *n = 1 at 12 hours; *n* = 6 at 16 and 18 hours; *n* = 7 at −24, 13.5, 20, and 22 hours. ^†^ *n* = 6 at 20 hours. ^‡^ *n* = 7 at 6, 13.5, and 24 hours. ^§^ *n* = 7 at −14 hours; *n* = 8 at − 12, 12, 13.5, and 16 hours. ^||^ *n* = 5 at −10.5, 0, and 22 hours. ^¶^ *n* = 4 at −10.5 and 1.5 hours. ^#^ *n* = 14 at 20 hours. ^Δ^ *n* = 12 at − 14 hours; *n* = 13 at − 12, −10.5, 1.5, 12, 13.5, and 16 hours. BID = twice daily; QD = once daily; rhPTH(1‐84) = recombinant human parathyroid hormone (1‐84).

At baseline, mean albumin‐corrected serum calcium levels were in the lower end of the reference range, or just below the reference range, in alignment with current guidelines for the management of hypoparathyroidism.^(^ ^2^, ^20^, ^21^ ^)^ In cohorts 1 and 2, in which patients received rhPTH(1‐84) without supplemental calcium, within‐cohort mean serum calcium concentrations were similar on day 1 and day −1 (when patients received conventional therapy only; Fig. 4*A*,*B*). In cohorts 3 and 4, which included supplemental calcium with rhPTH(1‐84) on day 1, within‐cohort mean serum calcium concentrations tended to be higher on day 1 compared with day −1 (Fig. 4*C*,*D*). No clear differences in mean albumin‐corrected serum calcium concentrations were observed among rhPTH(1‐84) treatments at 25 μg BID, 50 μg BID, and 100 μg QD (Fig. 4*E*).

**Fig. 5:**
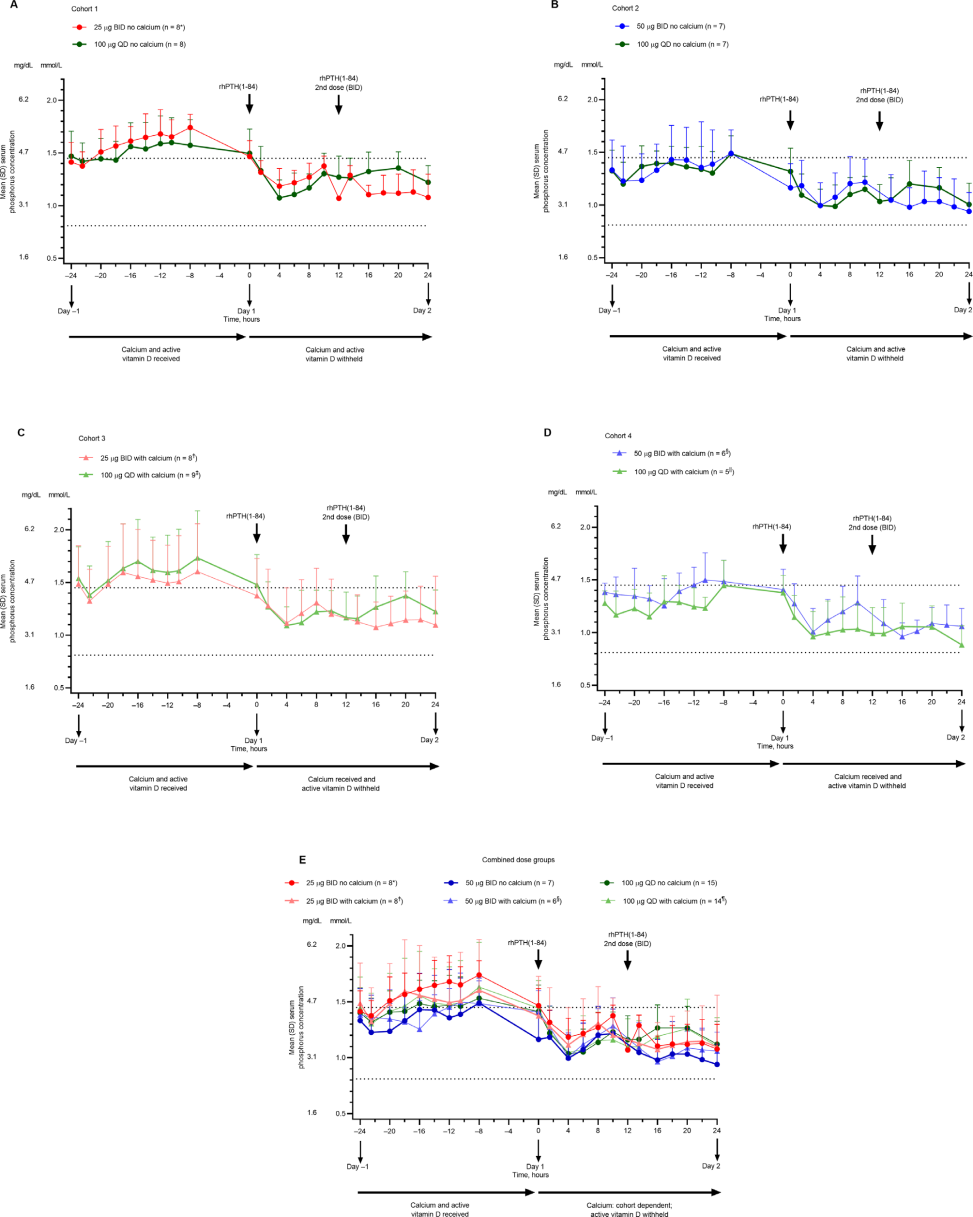
Mean serum phosphorus concentration in the study cohorts. (*A*) Cohort 1. (*B*) Cohort 2. (*C*) Cohort 3. (*D*) Cohort 4. (*E*) Combined dose groups (data for 100 μg QD without calcium supplementation are from cohorts 1 and 2, and data for 100 μg QD with calcium supplementation are from cohorts 3 and 4). Error bars represent SD. Horizontal lines indicate the reference range. *n = 1 at 12 hours; *n* = 6 at 16 and 18 hours; *n* = 7 at −24, 13.5, 20, and 22 hours. ^†^ *n* = 7 at 6 and 24 hours. ^‡^ *n* = 8 at −14 and 12 hours. ^§^ *n* = 5 at −10.5, 0, and 22 hours. ^||^ *n* = 4 at −10.5 and 1.5 hours. ^¶^ *n* = 13 at −14, −10.5, 1.5, and 12 hours. BID = twice daily; QD = once daily; rhPTH(1‐84) = recombinant human parathyroid hormone (1‐84).

In all cohorts, mean serum phosphorus concentrations were near or above the upper limit of the laboratory reference range on day −1 when patients were receiving their usual treatment of oral calcium and vitamin D (Fig. 5*A*–*D*). After administration of rhPTH(1‐84) at 25 μg BID, 50 μg BID, or 100 μg QD, serum phosphorus levels declined and were maintained within the reference range throughout day 1 (Fig. 5*E*). In cohorts 1, 2, and 3 (but not cohort 4), a possible trend was observed for greater reductions in serum phosphorus at hours 12 to 24 of day 1 with BID dosing versus QD dosing (Fig. 5*A*–*C*).

**Fig. 6:**
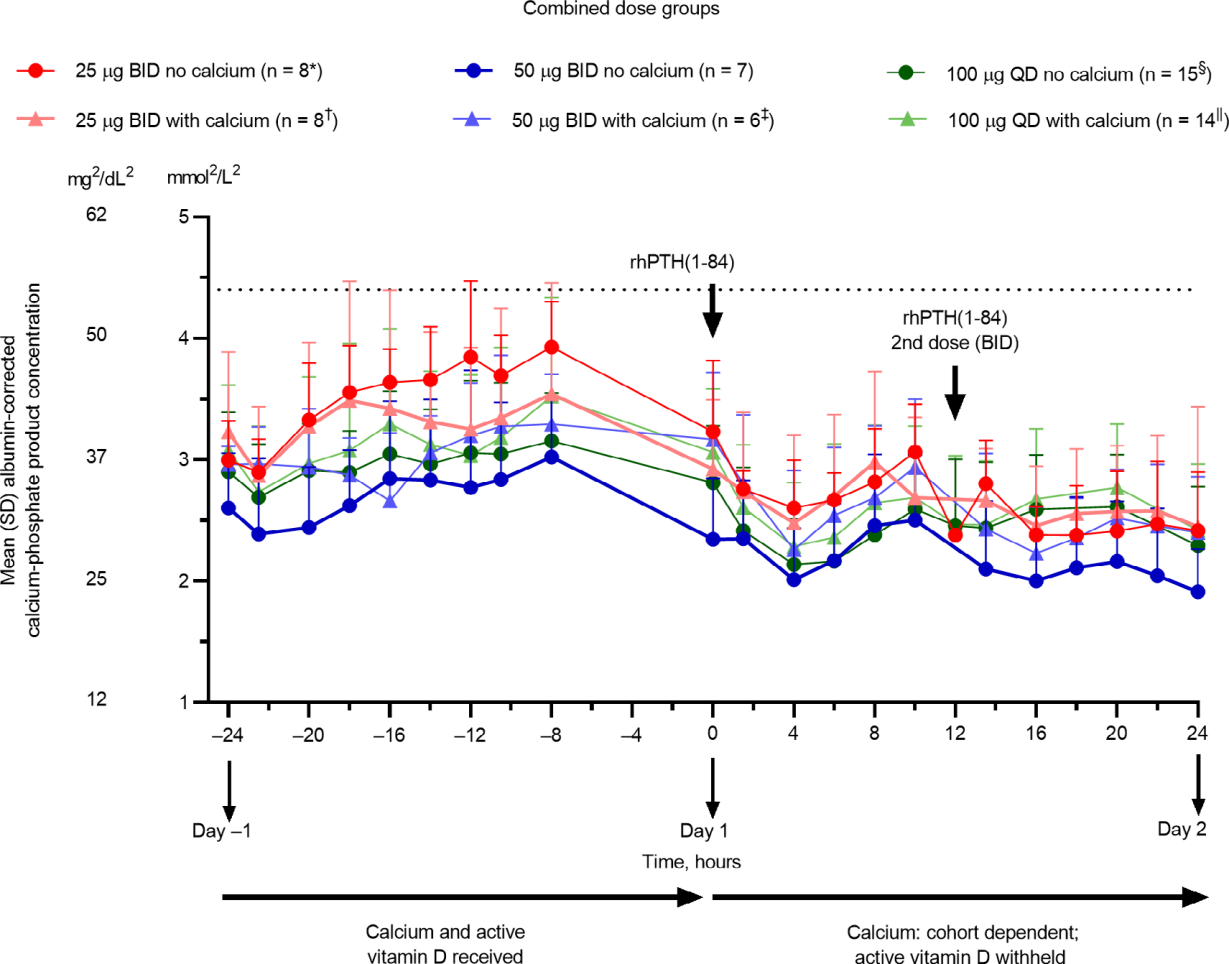
Mean albumin‐corrected serum calcium‐phosphate product concentration in the combined dose groups. Data for 100 μg QD without calcium supplementation are from cohorts 1 and 2, and data for 100 μg QD with calcium supplementation are from cohorts 3 and 4. Error bars represent SD. Horizontal lines indicate the upper safety limit. *n = 1 at 12 hours; *n* = 6 at 16 and 18 hours; *n* = 7 at −24, 13.5, 20, and 22 hours. ^†^ *n* = 7 at 6, 13.5, and 24 hours. ^‡^ *n* = 5 at −10.5, 0, and 22 hours. ^§^ *n* = 14 at 20 hours. ^||^ *n* = 12 at −14 hours; *n* = 13 at −12, −10.5, 1.5, 12, 13.5, and 16 hours. BID = twice daily; QD = once daily; rhPTH(1‐84) = recombinant human parathyroid hormone (1‐84).

On day 1, mean albumin‐corrected serum calcium‐phosphate product values were generally lower than those reported on day −1 (Fig. 6). A trend for reductions in mean albumin‐corrected serum calcium‐phosphate product values was observed with all rhPTH(1‐84) dose regimens, both with and without supplemental calcium. AUC_0–24_ for albumin‐corrected serum calcium‐phosphate values showed a numerical reduction on day 1 compared with day −1 (Table 4).

**Table 4:** Albumin‐Corrected Serum Calcium‐Phosphate Product PD Parameter and 24‐Hour Urinary PD Parameters by Treatment Group

	rhPTH(1‐84) treatment											
	No calcium						With calcium					
	25 μg BID (*n* = 9)		50 μg BID (*n* = 8)		100 μg QD (*n* = 17)		25 μg BID (*n* = 8)		50 μg BID (*n* = 8)		100 μg QD (*n* = 17)	
Parameter	Day −1	Day 1/day 2	Day −1	Day 1/day 2	Day −1	Day 1/day 2	Day −1	Day 1/day 2	Day −1	Day 1/day 2	Day −1	Day 1/day 2
Albumin‐corrected serum calcium‐phosphate product PD parameter												
AUC_0‐24_ (hours⋅mmol^2^/L^2^)	85.0 (7.09)^a^	64.4 (6.88)^b^	65.1 (12.5)^a^	52.5 (10.4)^a^	71.4 (9.18)^c^	58.6 (9.64)^c^	78.6 (17.0)	62.3 (14.6)^a^	74.7 (8.53)^d^	60.9 (10.5)^e^	76.8 (15.5)^f^	62.0 (11.9)^f^
24‐hour urinary PD parameters												
Calcium (mmol)	9.35 (3.96)^b^	4.90 (1.97)^a^	10.2 (3.71)^a^	5.14 (1.98)^a^	8.88 (3.74)^c^	6.04 (2.54)^f^	10.8 (2.83)	7.44 (2.54)	10.7 (2.92)^e^	8.81 (2.82)^e^	9.72 (2.94)^g^	7.93 (2.32)^f^
Calcium relative to creatinine (mmol/mmol)	0.806 (0.228)^b^	0.401 (0.167)^a^	0.836 (0.302)^a^	0.394 (0.128)^a^	0.727 (0.286)^c^	0.475 (0.173)^f^	0.874 (0.267)	0.678 (0.212)	0.855 (0.286)^e^	0.673 (0.238)^e^	0.807 (0.276)^g^	0.691 (0.236)^f^
Phosphorus (mmol)	29.0 (11.7)^b^	47.4 (10.9)^a^	20.1 (7.64)^a^	43.5 (9.26)^a^	22.1 (11.0)^c^	37.2 (8.94)^f^	21.0 (6.79)	37.3 (11.6)	19.9 (10.9)^e^	44.1 (13.9)^e^	20.4 (9.49)^g^	36.6 (14.3)^f^
Phosphorus relative to creatinine (mmol/mmol)	2.47 (0.725)^b^	3.73 (0.436)^a^	1.59 (0.487)^a^	3.42 (0.801)^a^	1.73 (0.618)^c^	2.99 (0.683)^f^	1.68 (0.526)	3.31 (0.701)	1.49 (0.465)^e^	3.29 (0.676)^e^	1.61 (0.597)^g^	3.04 (0.857)^f^
Magnesium (mmol)	4.25 (2.06)^b^	3.60 (0.884)^a^	4.26 (0.791)^a^	3.16 (0.973)^a^	4.47 (1.43)^c^	4.36 (1.13)^f^	4.34 (1.25)	3.98 (1.46)	4.77 (1.13)^e^	4.69 (1.83)^e^	4.65 (1.37)^g^	5.32 (1.85)^f^
Magnesium relative to creatinine (mmol/mmol)	0.354 (0.112)^b^	0.289 (0.069)^a^	0.344 (0.061)^a^	0.246 (0.069)^a^	0.360 (0.088)^c^	0.351 (0.096)^f^	0.354 (0.130)	0.356 (0.110)	0.382 (0.160)^e^	0.358 (0.166)^e^	0.399 (0.189)^g^	0.457 (0.161)^f^
cAMP (μmol)	2.31 (0.726)^a^	4.16 (0.708)^b^	2.37 (0.295)^a^	4.91 (0.822)^a^	2.57 (0.940)^c^	4.49 (0.997)^c^	2.23 (0.387)	3.47 (0.761)	2.81 (0.869)^e^	5.15 (1.25)^e^	2.52 (1.13)^g^	4.55 (1.15)^f^
cAMP relative to creatinine (μmol/mmol)	0.206 (0.072)^a^	0.355 (0.082)^b^	0.193 (0.028)^a^	0.384 (0.079)^a^	0.214 (0.081)^c^	0.371 (0.108)^c^	0.176 (0.032)	0.316 (0.064)	0.271 (0.061)^e^	0.398 (0.128)^e^	0.199 (0.065)^g^	0.396 (0.126)^f^
Citrate (mmol)	4.20 (1.08)^b^	5.12 (1.19)^a^	3.60 (1.17)^a^	4.24 (1.53)^a^	3.69 (1.29)^f^	3.62 (0.984)^f^	3.58 (1.22)	3.78 (1.39)	2.85 (0.542)^e^	3.62 (0.918)^e^	3.49 (1.20)^g^	3.53 (0.873)^f^
Citrate relative to creatinine (mmol/mmol)	0.390 (0.154)^b^	0.417 (0.138)^a^	0.296 (0.099)^a^	0.329 (0.115)^a^	0.314 (0.124)^f^	0.299 (0.106)^f^	0.291 (0.116)	0.345 (0.126)	0.232 (0.089)^e^	0.282 (0.096)^e^	0.302 (0.105)^g^	0.311 (0.098)^f^
Sodium (mmol)	231 (108)^b^	163 (66.7)^b^	144 (52.7)^a^	170 (56.2)^a^	167 (83.3)^c^	155 (50.4)^c^	218 (86.8)	169 (48.5)	260 (141)^e^	287 (68.3)^e^	252 (107)^f^	249 (107)^f^
Sodium relative to creatinine (mmol/mmol)	19.3 (5.96)^b^	13.7 (5.41)^b^	11.6 (3.71)^a^	13.1 (3.62)^a^	13.7 (6.91)^c^	12.8 (4.51)^c^	16.9 (5.46)	15.5 (4.25)	19.3 (7.20)^e^	23.7 (12.8)^e^	20.6 (6.24)^f^	22.0 (12.1)^f^
Albumin‐corrected serum calcium PD parameter												
AUC_0‐24_ (hours⋅mmol/L)	53.8 (5.28)^a^	52.7 (3.74)^b^	48.4 (2.45)^a^	48.9 (2.37)^a^	49.4 (2.84)^c^	49.3 (2.80)^c^	52.8 (3.49)^b^	54.0 (4.27)^a^	53.5 (3.10)^d^	54.5 (4.63)^e^	51.6 (3.84)^f^	53.4 (4.02)^f^
Serum phosphorus PD parameter												
AUC_0‐24_ (hours⋅mmol/L)	38.0 (3.23)^a^	29.3 (2.64)^b^	32.5 (6.07)^a^	25.8 (4.86)^a^	34.9 (4.61)^c^	28.5 (4.07)^c^	36.0 (8.61)^b^	27.9 (7.20)^a^	33.6 (4.61)^d^	26.9 (3.59)^e^	36.1 (7.74)^f^	27.9 (5.30)^f^

*Note*: Values are given as mean (SD) unless otherwise stated.

Abbreviation: AUC_0‐24_ = area under the curve from time 0 to 24 hours after the first rhPTH(1‐84) dose; BID = twice daily; day −1 = day before rhPTH(1‐84) treatment; day 1/day 2 = day of rhPTH(1‐84) treatment (ie, day 1) through 24 hours following the single QD rhPTH(1‐84) dose or the second BID rhPTH(1‐84) dose (ie, through 12 hours of day 2); *n* = number of patients; PD = pharmacodynamic; QD = once daily; rhPTH(1‐84) = recombinant human parathyroid hormone (1‐84).

^a^ *n* = 7.

^b^ *n* = 8.

^c^ *n* = 15.

^d^ *n* = 5.

^e^ *n* = 6.

^f^ *n* = 14.

^g^ *n* = 13.

**Fig. 7:**
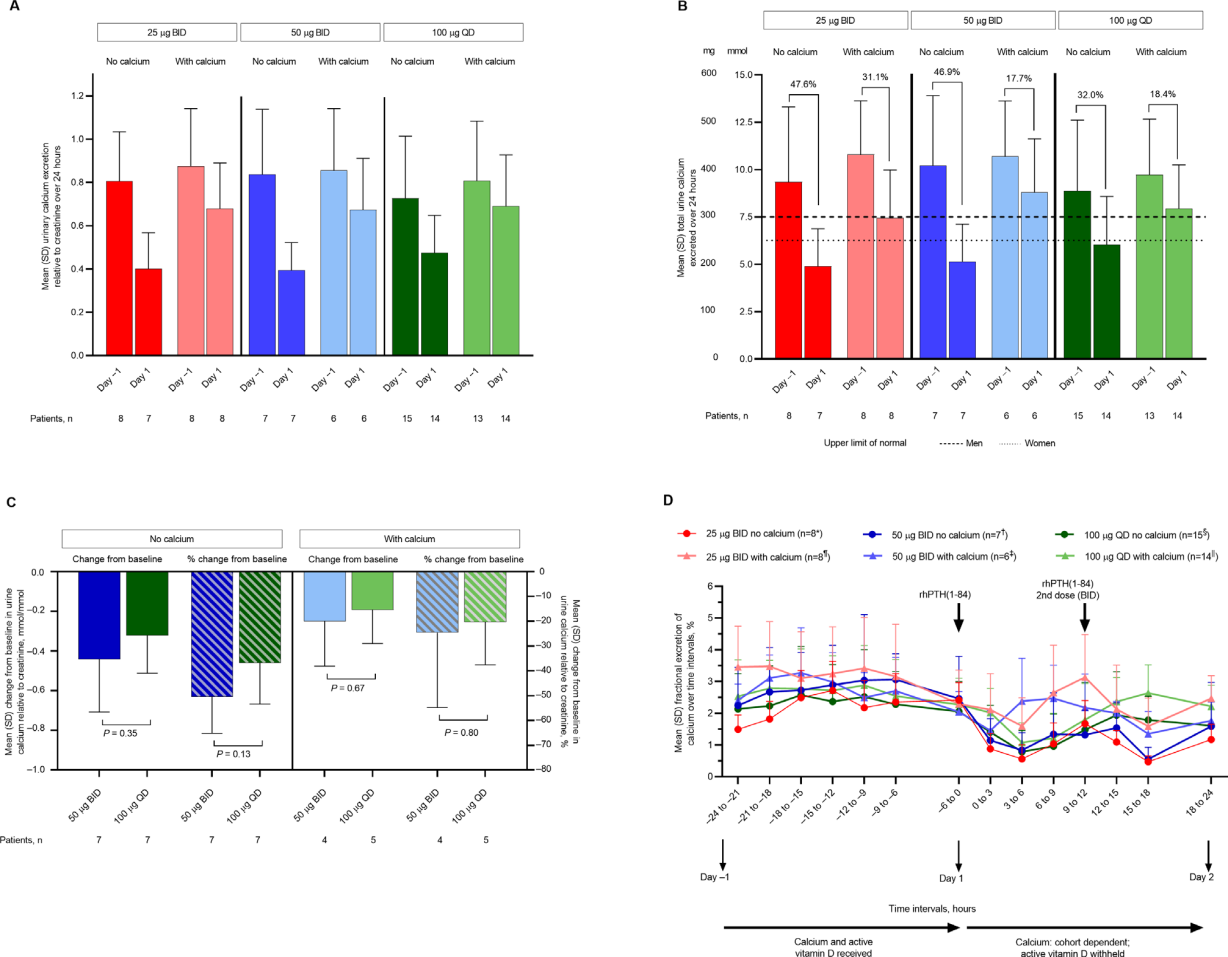
Mean urinary calcium excretion. (*A*) Urinary calcium excretion relative to creatinine over 24 hours. (*B*) Mean total urinary calcium excretion over 24 hours. (*C*) Change from baseline in urinary calcium relative to creatinine over 24 hours. (*D*) Fractional excretion of calcium over time. Data for 100 μg QD without calcium supplementation are from cohorts 1 and 2, and data for 100 μg QD with calcium supplementation are from cohorts 3 and 4. Error bars represent SD. In *B*, horizontal lines indicate the reference range. *n = 6 at 12 to 15 hours and 15 to 18 hours; *n* = 7 at 6 to 9, 9 to 12, and 18 to 24 hours. ^¶^ *n* = 7 at 12 to 15 hours. ^†^ *n* = 6 at −9 to −6 and 9 to 12 hours. ^‡^ *n* = 5 at −12 to −9 and − 9 to −6 hours. ^§^ *n* = 13 at 15 to 18 and 18 to 24 hours; *n* = 14 at 0 to 3, 3 to 6, 6 to 9, 9 to 12, and 12 to 15 hours. ^||^ *n* = 12 at −24 to −21, −12 to −9, and 15 to 18 hours; *n* = 13 at −21 to −18, −15 to −12, 0 to 3, 9 to 12, and 12 to 15 hours. BID = twice daily; QD = once daily.

Compared with conventional therapy with oral calcium and active vitamin D on day −1, all rhPTH(1‐84) dosing regimens with and without oral calcium decreased 24‐hour urinary calcium excretion relative to creatinine (Fig. 7*A*) and reduced total 24‐hour urinary calcium excretion (Fig. 7*B*). As expected, the rhPTH(1‐84)‐induced decrease in 24‐hour urinary calcium relative to creatinine was attenuated by adjunctive supplemental calcium (Table 4). Although no statistical testing was prespecified in this study, based on the observation of a numerical and incremental further reduction of urinary calcium excretion with BID dosing in comparison with QD dosing, a post hoc statistical analysis was performed to clarify whether the observed numerical differences between the 50 μg BID and 100 μg QD groups were statistically significant. This analysis revealed no nominally significant difference in the reduction of urinary calcium relative to creatinine between doses (Fig. 7*C*). In time course analyses, fractional excretion of calcium was lowest in most cohorts around 4 to 8 hours after rhPTH(1‐84) administration (Fig. 7*D*), showing a reduction from day −1.

**Fig. 8:**
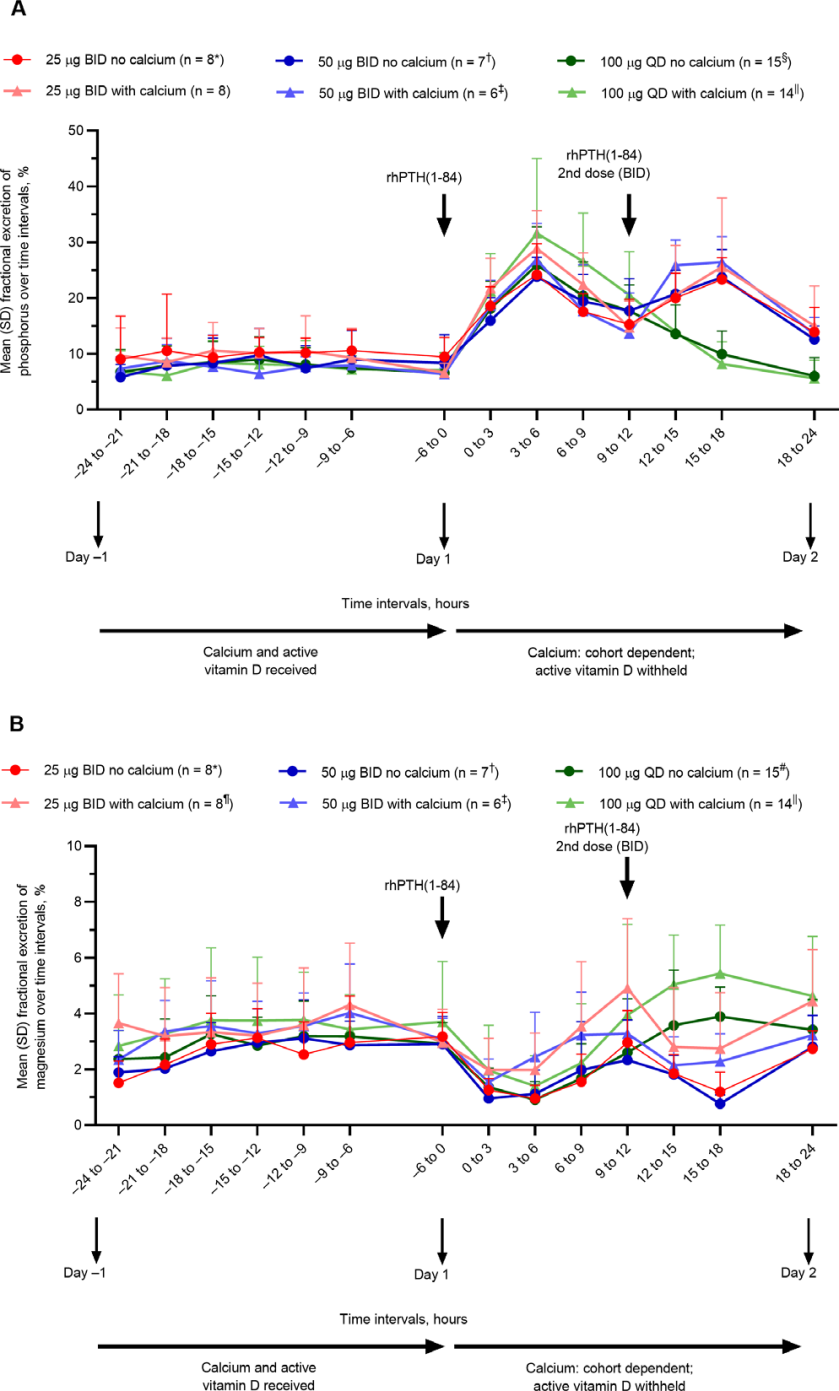
Fractional excretion per time interval of (*A*) phosphorus and (*B*) magnesium. Data for 100 μg QD without calcium supplementation are from cohorts 1 and 2, and data for 100 μg QD with calcium supplementation are from cohorts 3 and 4. Error bars represent SD. *n = 6 at 12 to 15 and 15 to 18 hours; *n* = 7 at 6 to 9, 9 to 12, and 18 to 24 hours. ^†^ *n* = 6 at −9 to −6 and 9 to 12 hours. ^‡^ *n* = 5 at −9 to −6 and −12 to −9 hours. ^§^ *n* = 13 at 15 to 18 hours; *n* = 14 at 0 to 3, 3 to 6, 6 to 9, 9 to 12, 12 to 15, and 18 to 24 hours. ^||^ *n* = 12 at −24 to −21, −12 to −9, and 15 to 18 hours; *n* = 13 at −21 to −18, −15 to −12, 0 to 3, 9 to 12, and 12 to 15 hours. ^¶^ *n* = 7 at 12 to 15 hours. ^#^ *n* = 13 at 15 to 18 and 18 to 24 hours; *n* = 14 at 0 to 3, 3 to 6, 6 to 9, 9 to 12, and 12 to 15 hours. BID = twice daily; QD = once daily; rhPTH(1‐84) = recombinant human parathyroid hormone (1‐84).

Administration of rhPTH(1‐84) with or without calcium supplementation under all dosing regimens markedly increased total 24‐hour urinary phosphorus compared with day −1 (Table 4). Fractional excretion of phosphorus reached a peak 3 to 6 hours after rhPTH(1‐84) administration, then gradually declined toward baseline through 18 to 24 hours postdose for QD injections (Fig. 8*A*). Similarly, a second peak was observed 3 to 6 hours after the second injection in the BID dosing groups. The magnitude of the changes was generally consistent across dosing regimens and was independent of adjunctive calcium supplementation. Total 24‐hour urinary excretion of magnesium tended to decrease after administration of rhPTH(1‐84) treatment, compared with day −1, except for the rhPTH(1‐84) 100 μg QD with calcium group, in which an increase in urinary magnesium after rhPTH(1‐84) was observed (Table 4). Fractional excretion of magnesium decreased between 0 and 6 hours after administration of rhPTH(1‐84) in all treatment groups, then began to increase gradually. For QD doses, fractional excretion of magnesium returned to or exceeded baseline values by 15 to 18 hours postdose (Fig. 8*B*). rhPTH(1‐84) administration stimulated urinary excretion of the second messenger cAMP in all dosing regimens, independent of adjunctive calcium supplementation (Table 4). No consistent change in urinary citrate or urinary sodium was observed after rhPTH(1‐84) administration (Table 4). Albumin‐corrected serum calcium remained relatively stable without calcium supplementation at all doses of rhPTH(1‐84) and numerically increased when rhPTH(1‐84) was administered in combination with calcium (Table 4). Serum phosphorus numerically decreased upon administration of rhPTH(1‐84) both with and without calcium, although this effect did not appear to be dose dependent (Table 4).

### Safety

Overall, 58 TEAEs were reported in 18 patients (52.9%; Table 5). Of the reported TEAEs, 45 were mild and 13 moderate in severity. No serious TEAEs were reported during the study. All but two of the reported TEAEs, pain in extremity (*n* = 1) and fatigue (*n* = 1), resolved by study completion. Among the reported TEAEs, 26 reported in 10 patients (29.4%) were considered by the investigator as related to treatment with rhPTH(1‐84). The most common TEAEs considered related to treatment were dizziness (14.5%; five events in five patients), nausea (11.8%; four events in four patients), and headache (8.8%; five events in three patients).

**Table 5:** Summary of TEAEs

	rhPTH(1‐84) treatment													
	No calcium						With calcium							
	25 μg BID (*n* = 9)		50 μg BID (*n* = 8)		100 μg QD (*n* = 17)		25 μg BID (*n* = 8)		50 μg BID (*n* = 8)		100 μg QD (*n* = 17)		Total (*N* = 34)	
Parameter	*n* (%)	m	*n* (%)	m	*n* (%)	m	*n* (%)	m	*n* (%)	m	*n* (%)	m	*n* (%)	m
Any TEAE	3 (33.3)	3	1 (12.5)	1	4 (23.5)	9	5 (62.5)	10	3 (37.5)	15	7 (41.2)	20	18 (52.9)	58
Nausea	0	0	0	0	3 (17.6)	3	1 (12.5)	1	2 (25.0)	2	1 (5.9)	1	7 (20.6)	7
Dizziness	0	0	0	0	3 (17.6)	3	1 (12.5)	1	0	0	2 (11.8)	2	6 (17.6)	6
Headache	0	0	0	0	0	0	1 (12.5)	1	1 (12.5)	5	3 (17.6)	4	4 (11.8)	10
Hot flush	0	0	0	0	0	0	2 (25.0)	2	0	0	2 (11.8)	2	4 (11.8)	4
Muscle spasms	1 (11.1)	1	0	0	0	0	0	0	1 (12.5)	1	1 (5.9)	1	2 (5.9)	3
Hypoesthesia	1 (11.1)	1	0	0	0	0	0	0	1 (12.5)	1	0	0	2 (5.9)	2
Tachycardia	0	0	0	0	0	0	1 (12.5)	1	0	0	1 (5.9)	1	2 (5.9)	2

Abbreviation: BID = twice daily; m = number of events; *n* = number of patients; QD = once daily; rhPTH(1‐84) = recombinant human parathyroid hormone (1‐84); TEAE = treatment‐emergent adverse event.

## Discussion

This study found no differences considered clinically meaningful in PD parameters in adult patients with chronic hypoparathyroidism treated with BID versus QD rhPTH(1‐84). The PK and PD characteristics of rhPTH(1‐84) have been assessed previously in an open‐label, phase I study that evaluated a single SC dose of rhPTH(1‐84) at 50 μg followed by 100 μg in patients with chronic hypoparathyroidism who were receiving calcium and vitamin D, using a design similar to that of the current study.^(^ ^18^ ^)^ Overall, PK results were broadly similar in the two studies. In both studies, a double PTH peak was observed after a single rhPTH(1‐84) injection. In the previous phase I study by Clarke et al., the initial PTH peak was observed a median of 10 to 15 minutes after injection and a second peak 1 to 3 hours later, with an estimated t_½_ of approximately 2.5 to 3 hours for both the 50 and 100 μg doses,^(^ ^18^ ^)^ which is similar to observations of the present study. A dose‐proportional increase in baseline‐adjusted AUC of PTH was observed between the 50 μg QD and 100 μg QD dose regimens in the previous phase I study,^(^ ^18^ ^)^ but was not clearly observed in the current study. Lack of PK dose proportionality in the current study was likely due to high variability in the PTH concentrations across cohorts, as evidenced by the high geometric mean coefficient of variation values for baseline‐adjusted PTH AUCs (ranging from 23% to 449%). In addition, some rhPTH(1‐84) dosing errors were suspected, which may have resulted in the observed lack of dose proportionality. Two patients receiving rhPTH(1‐84) 50 μg BID with calcium had unexpectedly low AUC_0–24_ values (<300 pg∙h/mL), a likely indicator of partial dosing.

In this study, albumin‐corrected serum calcium concentrations over 24 hours remained within levels recommended by treatment guidelines.^(^ ^2^, ^20^, ^21^ ^)^

Administration of rhPTH(1‐84), whether QD or BID, also resulted in a reduction of urinary calcium excretion. Coadministration of adjunctive calcium diminished the effect of rhPTH(1‐84) on urinary calcium decline, likely because of increased renal filtered load of calcium. Indeed, cohorts that received supplemental calcium showed a trend for higher levels of serum calcium after rhPTH(1‐84) injection compared with cohorts that received no supplemental calcium. For this study, the sample size was not based on statistical power considerations because the statistical analyses were primarily descriptive, and no hypothesis testing was prespecified in the study protocol. This study was not designed to show a significant effect of 50 μg BID versus 100 μg QD rhPTH(1‐84), and none of the post hoc analyses suggested a significant effect of a 50 μg BID rhPTH(1‐84) dose to reduce urinary calcium excretion compared with a 100 μg QD dose, although there appeared to be a further numerically incremental reduction in urinary calcium excretion with BID administration compared with QD. As an estimate only, a post hoc power analysis suggested that, with the current sample size and observed treatment difference in this study (11.4% without calcium and 0.8% with calcium, respectively), the power to detect a statistically significant difference without calcium was 18% and with calcium 2.7%. That same analysis suggested that, to detect a statistically significant difference at an assumed treatment difference of 20%, with a superiority design without calcium, a sample size of 15 to complete would have been required, greater than the sample size within the study. Similarly, a sample size estimation yields an *n* of 29 for a superiority study with calcium assuming the same treatment difference of 20% and 80% power. In this study, rhPTH(1‐84) administration caused a rapid reduction of urinary calcium excretion, but no inference regarding the effect of long‐term treatment is possible. It is anticipated that long‐term treatment may be necessary to normalize urinary calcium. After 24 weeks of treatment in the phase III REPLACE study, urinary calcium excretion had declined from baseline in both the rhPTH(1‐84) and placebo arms but remained above the reference range for women in the rhPTH(1‐84) arm at the end of the study. No significant differences in 24‐hour urinary calcium excretion were observed between the two arms at the end of the study.^(^ ^16^, ^17^, ^22^ ^)^ However, in two long‐term, single‐arm, open‐label clinical trials of rhPTH(1‐84) treatment, mean urinary calcium excretion decreased from above the reference range at baseline to within the reference range with 5 to 8 years of treatment.^(^ ^17^, ^23^ ^)^

In patients with chronic hypoparathyroidism, the lack of phosphaturic PTH effects can result in hyperphosphatemia. Increased serum phosphorus has been reported to be associated with vascular stiffness^(^ ^24^ ^)^ and vascular calcification,^(^ ^25^, ^26^ ^)^ which are independent predictors of cardiovascular disease in the general population.^(^ ^27^, ^28^, ^29^ ^)^ In this study, administration of rhPTH(1‐84) increased urinary phosphorus excretion in all cohorts and lowered serum phosphorus concentrations. These effects were independent of oral calcium supplementation. Larger and longer studies are required to confirm these findings and to understand the clinical significance of these differences between dosing frequencies.

With respect to other urinary PD parameters, a numerical increase in urinary cAMP excretion was observed with rhPTH(1‐84) administration, under all treatment regimens. Conversely, inconsistent results were seen for urinary sodium and urinary citrate across rhPTH(1‐84) treatments. The urinary citrate results with short‐term rhPTH(1‐84) reported here are in contrast to data from Gafni and colleagues^(^ ^30^ ^)^ showing a significant decrease from baseline in urinary citrate after 6 months of synthetic PTH(1‐34) administration, an effect that persisted through 5 years of treatment. In Gafni and colleagues,^(^ ^30^ ^)^ at the last measure after treatment with human PTH 1–34 (up to 5 years), urinary pH was significantly increased and blood bicarbonate concentration was significantly decreased. These findings suggest a mild metabolic acidosis,^(^ ^30^, ^31^ ^)^ which is a key determinant of urinary citrate excretion.^(^ ^32^ ^)^ In the present study, which was shorter than the study by Gafni and colleagues^(^ ^30^ ^)^ and evaluated rhPTH(1‐84), there was no meaningful change in serum bicarbonate and a modest increase in urinary pH. There was a numerical reduction in 24‐hour urinary magnesium excretion in all but one treatment regimen in this study (100 μg QD with calcium), but time course data revealed a more nuanced pattern. Fractional excretion of magnesium tended to decrease during the first 6 hours after rhPTH(1‐84) injection but then began to increase back to or beyond baseline levels.

No new safety findings were observed with rhPTH(1‐84), whether administered QD or BID, in patients with chronic hypoparathyroidism. In addition, although dosing regimens that included adjunctive calcium generally resulted in an increased albumin‐corrected serum calcium concentration when compared with regimens without adjunctive calcium, albumin‐corrected serum calcium concentrations remained in the lower half or slightly below the reference range for all treatment groups in this study.

This study has several limitations. Data were generated for a single day of QD or BID dosing, and results cannot be generalized to long‐term treatment strategies. Because of the large blood volume needed for the PK evaluations, only two rhPTH(1‐84) treatments for PK and PD assessment within a month could be performed in any given participant. Consequently, rhPTH(1‐84) dose regimens 25, 50, and 75 μg QD, and 75 and 100 μg BID were not investigated. However, patients in all cohorts received the 100 μg QD treatment and either a 25 or 50 μg BID treatment. The study was also limited by small cohort sample sizes, which have the potential for type II errors in statistical analysis.^(^ ^33^ ^)^ In addition, sequential enrollment of study cohorts, potentially resulting in PTH assay variability over the duration of the study, likely contributed to observed PK variability. There were also differences in BMI across cohorts that may have affected PK and PD parameters. Furthermore, PD effects should be interpreted with caution because of the complexity of factors, such as bone turnover and kidney function, that contribute to calcium homeostasis. Finally, although active vitamin D intake was paused on the day of rhPTH(1‐84) dosing, some biological activity likely persisted. A study including longer treatment durations would be needed to confirm the results reported here.

Our study population, although modest, is generally representative of patients with hypoparathyroidism, with the majority of patients included being women above the age of 45 years.^(^ ^34^ ^)^ Short‐term treatment with rhPTH(1‐84) in this study maintained serum calcium, lowered serum phosphorus, decreased urinary calcium excretion, and increased urinary phosphorus excretion. Overall, no substantial differences in PD parameters were observed with BID versus QD rhPTH(1‐84) dosing. Although these observations were similar both with and without calcium supplementation, the data on urinary calcium excretion suggest that administration of rhPTH(1‐84) without calcium supplementation may be optimal; however, these results pertain to one day of treatment only, thus, data on long‐term treatment should also be considered when evaluating the most effective treatment regimen. No new safety findings were observed with rhPTH(1‐84), whether administered QD or BID, in patients with chronic hypoparathyroidism. Further studies would be needed to better understand the effects of alternative rhPTH(1‐84) dosing strategies on serum and urine biochemistry in patients with chronic hypoparathyroidism.

## Author Contributions

**Steven W. Ing:** Conceptualization; investigation; methodology; writing – original draft; writing – review and editing. **Richard D. Finkelman:** Conceptualization; investigation; methodology; writing – original draft; writing – review and editing. **Ping He:** Formal analysis; methodology; writing – original draft; writing – review and editing. **Aliya A. Khan:** Conceptualization; investigation; methodology; writing – original draft; writing – review and editing. **Michael Mannstadt:** Conceptualization; investigation; methodology; writing – original draft; writing – review and editing. **Lars Rejnmark:** Conceptualization; investigation; methodology; writing – original draft; writing – review and editing. **Ivy Song:** Conceptualization; formal analysis; methodology; writing – original draft; writing – review and editing. **István Takács:** Conceptualization; investigation; methodology; writing – original draft; writing – review and editing. **Yuna Wu:** Formal analysis; methodology; writing – original draft; writing – review and editing.

## Disclosures

SWI has served as an advisory board member and research investigator for Takeda Pharmaceuticals USA, Inc., and as a research investigator for Amgen Inc., Chugai Pharmaceutical Co., Ltd, Radius Health Inc., and Ultragenyx Pharmaceutical. R.D.F. was an employee of Takeda Pharmaceuticals USA, Inc. at the time the research was conducted and is now a consultant for Takeda Pharmaceuticals USA, Inc. with stock options. I.S. and Y.W. are employees of and hold stock/stock options at Takeda Pharmaceuticals USA, Inc. P.H. was an employee of Takeda Pharmaceuticals USA, Inc., at the time the research was conducted and is now an employee of Travere Therapeutics, San Diego, CA, USA. A.A.K. has served as a research investigator for Takeda Pharmaceuticals USA, Inc. M.M. has served as an advisory board member, consultant, and research investigator for Takeda Pharmaceuticals USA, Inc., as a consultant and research investigator for Calcilytix and Chugai Pharmaceutical Co., Ltd, and as a consultant for Amolyt Pharma. L.R. has served as an advisory board member, research investigator, and speaker for and received research support from Takeda Pharmaceuticals USA, Inc.; L.R. has also served as an advisory board member for Amolyt Pharma and a research investigator for Ascendis Pharma. I.T. has served as a research investigator and received research support from Takeda Pharmaceuticals USA, Inc.

### Peer Review

The peer review history for this article is available at https://www.webofscience.com/api/gateway/wos/peer‐review/10.1002/jbm4.10758.

## Data Availability

The datasets, including the redacted study protocol, redacted statistical analysis plan, and individual participants' data supporting the results reported in this article, will be made available within 3 months from initial request, to researchers who provide a methodologically sound proposal. The data will be provided after its de‐identification, in compliance with applicable privacy laws, data protection, and requirements for consent and anonymization.
